# Surgery of the aortic root: should we go for the valve-sparing root reconstruction or the composite graft-valve replacement is still the first choice of treatment for these patients?

**DOI:** 10.5935/1678-9741.20150028

**Published:** 2015

**Authors:** Fernando de Azevedo Lamana, Ricardo Ribeiro Dias, Jose Augusto Duncan, Leandro Batisti de Faria, Luiz Marcelo Sa Malbouisson, Luciano de Figueiredo Borges, Charles Mady, Fábio Biscegli Jatene

**Affiliations:** 1 Medicine student at Universidade Federal de Minas Gerais (UFMG), Belo Horizonte, MG, Brazil.; 2 Cardiomiopathies and Aorta Diseases Surgical Center at Instituto do Coração of Hospital das Clínicas of Faculdade de Medicina da Universidade de São Paulo (InCor HC-FMUSP), São Paulo, SP, Brazil.; 3 Instituto do Coração of Hospital das Clínicas of Faculdade de Medicina da Universidade de São Paulo (InCor HC-FMUSP), São Paulo, SP, Brazil.; 4 Anestesiology Department of Hospital das Clínicas of Faculdade de Medicina da Universidade de São Paulo (HC-FMUSP), São Paulo, SP, Brazil.; 5 Departament of Morfology of Instituto de Ciências Biológicas of Universidade Federal de Minas Gerais (UFMG), Belo Horizonte, MG, Brazil.

**Keywords:** Aorta, Thoracic, Aortic Aneurysm, Aortic Aneurysm, Thoracic, Aortic Diseases, Cardiovascular Surgical Procedures

## Abstract

**Objective:**

To compare the results of the root reconstruction with the aortic valve-sparing operation versus composite graftvalve replacement.

**Methods:**

From January 2002 to October 2013, 324 patients underwent aortic root reconstruction. They were 263 composite graft-valve replacement and 61 aortic valve-sparing operation (43 reimplantation and 18 remodeling). Twenty-six percent of the patients were NYHA functional class III and IV; 9.6% had Marfan syndrome, and 12% had bicuspid aortic valve. There was a predominance of aneurysms over dissections (81% vs. 19%), with 7% being acute dissections. The complete follow-up of 100% of the patients was performed with median follow-up time of 902 days for patients undergoing composite graft-valve replacement and 1492 for those undergoing aortic valve-sparing operation.

**Results:**

In-hospital mortality was 6.7% and 4.9%, respectively for composite graft-valve replacement and aortic valve-sparing operation (ns). During the late follow-up period, there was 0% moderate and 15.4% severe aortic regurgitation, and NYHA functional class I and II were 89.4% and 94%, respectively for composite graft-valve replacement and aortic valve-sparing operation (ns). Root reconstruction with aortic valve-sparing operation showed lower late mortality (*P*=0.001) and lower bleeding complications (*P*=0.006). There was no difference for thromboembolism, endocarditis, and need of reoperation.

**Conclusion:**

The aortic root reconstruction with preservation of the valve should be the operation being performed for presenting lower late mortality and survival free of bleeding events.

## Abstract (pt)

**Objetivo:**

Analisar comparativamente os resultados da operação de preservação da valva aórtica e do tubo valvulado nas reconstruções da raiz da aorta.

**Métodos:**

No período de janeiro de 2002 a outubro de 2013, 324 pacientes foram submetidos à reconstrução da raiz da aorta. Foram 263 tubos valvulados e 61 preservações da valva aórtica (43 reimplantes e 18 remodelamentos). 26% dos pacientes estavam em classe funcional III e IV; 9,6% com síndrome de Marfan e 12% apresentavam valva aórtica bivalvulada. Houve predomínio dos aneurismas sobre as dissecções (81% contra 19%), sendo 7% de dissecções agudas. O seguimento completo de 100% dos pacientes foi realizado com tempo mediano de seguimento de 902 dias para pacientes submetidos à tubo valvulado e de 1492 para aqueles submetidos à preservação da valva aórtica.

**Resultados:**

A mortalidade hospitalar foi de 6,7% contra 4,9% respectivamente para tubo valvulado e preservação da valva aórtica (ns). No seguimento tardio, a insuficiência aórtica importante foi de 0% e 5,8%, e a insuficiência cardíaca crônica, classe funcional I e II de 89,4% e 94%, respectivamente, para tubo valvulado e preservação da valva aórtica (ns). A reconstrução da raiz da aorta com a preservação da valva aórtica apresentou menor mortalidade tardia (*P*=0,001) e menos complicações hemorrágicas (*P*=0,006). Não houve diferença para tromboembolismo, endocardite ou necessidade de reoperação.

**Conclusão:**

A reconstrução da raiz da aorta com a preservação valvar deve ser a operação a ser realizada por apresentar menor mortalidade e sobrevida livre de eventos hemorrágicos.

**Table:** 

**Abbreviations, acronyms & symbols**	
ACC	American College of Cardiology
AHA	American Heart Association
AVS	Aortic valve-sparing operation
CVR	Composite graft-valve replacement
NYHA	New York Heart Association

## INTRODUCTION

The choice of treatment for the correction of diseases affecting the aortic root and aortic valve is its replacement for a valved conduit. However, there is a large amount of discussion about which is the best valved conduit (homograft, xenograft, autograft, mechanical valve and prosthetic conduit or bioprosthetic) and the variety of possibilities according to different age groups^[1]^. The possibilities for aortic root reconstruction with preservation of the aortic valve, regardless of the degree of aortic regurgitation, has been largely documented albeit with several considerations about its non-applicability to every patient as well as its complexity, making it difficult to be used by all surgeons^[2-4]^.

In an attempt to follow the advances in aortic surgery presented worldwide, we have performed many of the procedures proposed with results similar or very close to the ones presented, especially in reference centers^[3-5]^.

Aortic root operations have low mortality rates and patients who have undergone the procedure show the same life expectancy and quality of life as the healthy population of the same age group^[6]^. Thus, these patients need to be identified and treated.

In our midst, we still have little knowledge on the impact of this disease in the general population. It has been observed that its mortality in the state of São Paulo is still very high due to either the shortcomings and inefficiency of our health system in identifying, screening, and treating these patients or the inadequate results obtained with hospitalized patients receiving drug treatment or even those who had undergone a surgical procedure (despite results being substantially better when these patients underwent surgery)^[7]^.

A first step has been taken. In the state of São Paulo, even though a significant increase has been observed in the number of diagnostics, hospitalizations, and procedures during the period of the study^[7]^, there is a lot to be done in order to improve the results of these interventions.

In a system where the population still has limited access to healthcare, procedures that allow simpler follow-up should always be pursued as long as their results are similar to those of conventional treatment.

To this end, this study sets out to evaluate whether valve-sparing root reconstructions show similar or better results to those obtained with composite graft-valve replacement, especially when comparing similar populations of patients.

## METHODS

Between January 2002 and October 2013, 324 patients underwent surgery for aortic root reconstruction. Among them, 263 patients underwent composite graft-valve replacement (CVR) (251 mechanical prosthetic valves and 12 bioprosthetic valves) and 61 patients underwent aortic valve-sparing operation (AVS) (43 reimplantation and 18 remodeling).

The indication for surgery was in accordance with the ACC/AHA guidelines^[8]^.

A retrospective data analysis was performed by searching the institution's database as well as by talking to individual patients on the phone, when needed.

The study was approved by the Research Ethics Committee of the institution and informed consent was deemed unnecessary due to the study characteristics.

The patients who underwent aortic root reconstruction were predominantly male (73%), with a mean age of 52 years and predominance of aneurysms over dissections (81% vs. 19%), of which 7% were acute dissections. Mean diameter was 5.7cm, mean body mass index was 25, and ventricular function was almost fully preserved.

Among the 31 patients who had Marfan syndrome (9.5%), 19 were submitted to CVR and 12 to AVS. The other 39 patients who had bicuspid aortic valve (12%), 38 underwent CVR and 1 AVS.

There were 62 reoperations (19%), every single one of them was CVR; 37 were reoperations of previous cardiac surgery (60%) and 25 were reoperations of aortic surgery. In addition, there were 76 associated procedures (23.4%); 54 of them during CVR surgery (71%).

Table 1 shows the comparison of demographic characteristics of patients submitted to aortic root reconstruction, before and after the groups were matched according to the propensity score.

**Table 1:** Preoperative characteristics of patients from the original CVR and AVS groups and propensity score matched groups.

	Original cohort			Propensity score matched cohort		
	CVR	AVS	*P* value	CVR	AVS	*P* value
Variables	n=263	n=61		n=60	n=61	
Mean age, years (mean ± SD)	55±15	48±15	0.02	58±14	48±15	0.001
Male, n (%)	19 (73.8)	42 (68.9)	0.43	43 (71.7)	42 (68.9)	0.73
BMI,Kg/m^2^ (mean ± SD)	25.8±4.5	24,1±4.6	0.03	25.3±4	24.1±4.6	0.25
LVEF, % (mean ± SD)	0.57±0.12	0.61±0.1	0.03	0.59±0.13	0.61±0.1	0.37
LVEDV, mL (mean ± SD)	211±85	222±108	0.48	233±89	222±108	0.6
Aortic diameter, mm (mean ± SD)	58±11	56±8	0.32	57±11	56±8	0.51
						
Risk factor						
Marfan syndrome, n (%)	19 (7.2)	12 (19.7)	0.003	3 (5)	12 (19.7)	0.02
Bicuspid Aortic valve, n (%)	38 (14.4)	1 (16)	0.004	3 (5)	1 (16)	0.36
Hypertension, n (%)	175 (66.5)	45 (73.8)	0.27	46 (76.7)	45 (73.8)	0.71
Diabetes mellitus, n(%)	18 (6.8)	3 (4.9)	0.77	3 (5)	3 (5)	0.99
Dyslipidemia, n(%)	55 (20.9)	10 (16.4)	0.42	11 (18.3)	10 (16.4)	0.77
Renal failure, n (%)						
ARF, n (%)	6 (2.3)	2 (3.3)	0.65	1 (1.7)	2 (3.3)	0.99
CRF, n (%)	21 (8)	2 (3.3)	0.27	6 (10)	2 (3.3)	0.16
Dialytic renal failure, n (%)	0 (0)	1 (16)	0.18	0 (0)	1 (16)	0.31
Smoking n (%)	88 (33.5)	24 (39.3)	0.38	20 (33.3)	24 (39.3)	0.49
Family history, n (%)	20 (7.6)	7 (11.5)	0,32	4 (6.7)	7 (11.5)	0.52
COPD, n (%)	19 (7.2)	1 (16)	0.14	6 (10)	1 (16)	0.06
CVA, n (%)	7 (2.7)	0 (0)	0.35	2 (3.3)	0 (0)	0.24
Cancer, n (%)	5 (1.9)	0 (0)	0.58	1 (16)	0 (0)	0.49
HIV+, n (%)	6 (2.3)	0 (0)	0.59	0 (0)	0 (0)	-
Coronary insufficiency, n (%)	44 (16.7)	8 (13.1)	0.48	12 (20)	8 (13.1)	0.3
AMI, n (%)	14 (5.3)	1 (16)	0.32	2 (3.3)	1 (1.7)	0.61
Chest pain, n (%)	91 (34.6)	22 (36.1)	0.82	12 (20)	22 (36.1)	0.05
Reoperation, n (%)	62 (23.8)	0 (0)	<0.001	11 (18.6)	0 (0)	<0.001
						
Heart failure, n (%)			0.02			0.03
FC I	132 (50.2)	38 (62.3)		26 (43.3)	38 (62.3)	
FC II	56 (21.3)	15 (24.6)		18 (30)	15 (24.6)	
FC III	53 (20.2)	6 (9.8)		14 (23.3)	6 (9.8)	
FC IV	22 (8.4)	2 (3.3)		2 (3.3)	2 (3.3)	
						
Indication for surgery, n (%)						
Aneurysm	204 (77.6)	58 (95.1)	0.002	52 (86.7)	58 (95.1)	0.12
Acute type A aortic dissection	18 (6.9)	0 (0)	0.02	1 (16)	0 (0)	0.49
Chronic type A aortic dissection	47 (17.9)	4 (6.6)	0.03	7 (11.7)	4 (6.6)	0.36
						
Function of aortic valve, n (%)			0.03			0.29
Normal	16 (6.3)	5 (8.3)		7 (11.7)	5 (8.3)	
Minimal AI	3 (1.2)	2 (3.3)		0 (0)	2 (3.3)	
Mild AI	47 (17.9)	16 (26.7)		6 (10)	16 (26.7)	
Moderate AI	68 (26.6)	18 (30)		22 (36.7)	18 (30)	
Severe AI	122 (47.7)	19 (31.7)		25 (41.7)	19 (31.7)	
						
Urgent operation	97 (36.7)	0 (0)	<0.001	0 (0)	0 (0)	-

CVR=composite graft-valve replacement; AVS=aortic valve-sparing operation; SD=standard deviation; BMI=Body Mass Index; LVEF=left ventricle ejection fraction; LVEDV=left ventricle enddiastolic volume; ARF=acute renal failure; CRF=chronic renal failure; COPD=chronic obstructive pulmonary disease; CVA=cerebrovascular accident; HIV+=positive status for human immunodeficiency vírus; AMI=acute myocardial infarction; FC=functional class (NYHA); AI=aortic insufficiency

Patients submitted to aortic root reconstruction in connection with complex thoracic aorta procedures (approach of more than three thoracic aorta segments in a single procedure) were excluded from the study.

### Surgical technique

Surgery was performed through median sternotomy when the aortic disease was restricted to the aortic root/ascending aorta and the arterial line was established in the aortic arch (212 patients/65%). In patients where an approach of the aortic arch was necessary, whenever the brachiocephalic trunk was uncompromised it served as the site of the arterial line (91 patients/28%). In cases of acute proximal dissection, there was cannulation of the right subclavian artery (21 patients/6.5%). Cerebral protection was achieved with hypothermia at 25ºC and selective cerebral perfusion via a carotid artery associated with topical hypothermia and thiopental sodium.

Venous drainage was performed preferably through a single two-stage cannula in the right atrium (except when it was necessary to approach the mitral valve) and drainage of the left chambers was done through a catheter placed in the left ventricle via the right superior pulmonary vein.

Myocardial protection, initially performed with antegrade intermittent cold cardioplegia (exclusively up to 2009), has been increasingly used in less complex procedures (80.2% CVR) and replaced by histidine-tryptophan-ketoglutarate solution (Custodiol^®^ HTK) in more extensive procedures (19.8% CVR and 24.6% AVS).

Systemic perfusion temperature for procedures in which the aortic arch had to be approached (partial approach) was 25ºC. When the approach was deemed unnecessary, moderate hypothermia was kept at 32ºC.

In the aortic root reconstructions performed with CVR, mean CPB and myocardial ischemia time were 136 and 108 minutes, respectively. In AVS procedures, mean times were 158 and 135 minutes, respectively (Table 2).

**Table 2:** Intraoperative data of patients from the original CVR and AVS groups and propensity score matched groups.

	Original cohort				Propensity score matched cohort		
	CVR	AVS	*P* value		CVR	AVS	*P* value
Variables	n=263	n=61			n=60	n=61	
CPB time, min (mean±SD)	136±38	158±31	<0.001		132±29	158±31	<0.001
Ischemic time, min (mean±SD)	108±30	135±25	<0.001		100±22	135±25	<0.001
							
Aortic approach, n (%)			<0.001				<0.001
Bentall	225 (85.6)	0 (0)			56 (93.3)	0 (0)	
Cabrol	38 (14.4)	0 (0)			4 (6.7)	0 (0)	
Reimplantation	0 (0)	43 (70.5)			0 (0)	43 (70.5)	
Remodeling	0 (0)	18 (29.5)			0 (0)	18 (29.5)	
							
Arterial line, n (%)			0.13				0.39
CPB	161 (61.2)	51 (83.6)			35 (58.3)	51 (83.6)	
CPB + TCA + RCP	1 (0.4)	0 (0)			1 (1.7)	0 (0)	
Femoro-femoral CPB	3 (1.1)	0 (0)			0 (0)	0 (0)	
Subclavian + 1 carotid	19 (7.2)	1 (16)			2 (3.3)	1 (16)	
Subclavian + 2 carotids	1(0.4)	0 (0)			0 (0)	0 (0)	
BCT + 1 carotid	68 (25.9)	9 (14.8)			21 (35)	9 (14.8)	
BCT + 2 carotids	9 (3.4)	0 (0)			1 (1.7)	0 (0)	
BCT + SCP via 2 carotids	1 (0.4)	0 (0)			0 (0)	0 (0)	
							
Associated procedures, n (%)							
MR	35 (13.3)	6 (9.8)	0.46		7 (11.7)	6 (9.8)	0.74
MiVR/plasty	13 (4.9)	16 (16.4)	0.002		1 (1.7)	16 (16.4)	0.008
Descending Aorta stent gafting	3 (1.1)	0 (0)	0.4		3 (5)	0 (0)	0.11
Descending Aorta conduit	3 (1.1)	0 (0)	0.4		0 (0)	0 (0)	-

CVR=composite graft-valve replacement; AVS=aortic valve-sparing operation; SD=standard deviation; CPB=cardiopulmoray bypass; SCP=selective cerebral perfusion; BCT=brachiocephalic trunk; TCA=total circulatory arrest; RCP=retrogade cerebral perfunsion; MR=myocardial revascularization; MiVR=mitral valve replacement

### Follow-up

Information on these patients was continuously gathered from the results of outpatient follow-up and telephone contact with patients from other regions.

Patients were evaluated for events related to prolonged use of oral anticoagulants, thromboembolic and hemorrhagic events, infection of prosthetic valve, endocarditis with or without the need for reoperation, and reoperation for any reason. Ecchymosis, conjunctival hemorrhage, epistaxis, hematuria, larger bleedings and those with hemodynamic repercussions (cardiac tamponade, upper gastrointestinal bleeding or enterorrhagia), as well as bleeding to the central nervous system were considered as minor hemorrhagic events.

Complete follow-up of 100% of the patients was performed with a median time of 902 days [213 (25^th^ percentile) - 1757 (75^th^ percentile)] for patients who underwent CVR and 1492 days [487 (25^th^ percentile) - 2385 (75^th^ percentile)] for those who underwent AVS. Follow-up period ended in October 2013.

### Statistical Analysis

The results were expressed as mean ± SD and percentages. For the analysis, normal distribution was confirmed by the Kolmogorov-Smirnov test and visual analysis of the data. Continuous variables were compared using the Student t-test for pairwise comparisons and the Wilcoxon test for unpaired data. Categorical variables were assessed through either Chi-square or Fischer's exact test. Kaplan-Meier curves and the log-rank test were used to compare survival rates in the AVS and CVR groups. Values of *P*<0.05 were considered statistically significant. All analyses were performed using IBM - SPSS (version 21, IBM Corp Armonk, NY).

### Propensity score

In order to reduce selection bias resulting from the collection of non-randomized data from distinct periods of time as well as to balance the sample characteristics, patients from the AVS and CVR groups were propensity matched based on the estimated probability of being treated. The procedure consists of matching patients from the intervention group (AVS) with similar characteristics to those of the control group (CVR). First, a logistic regression model was created using the group as the dependent variable. The most relevant confounders (age, left ventricular ejection fraction, degree of aortic regurgitation, and congestive heart failure according to the NYHA classification) were inserted as predictors and the confidence level for corresponding tolerance intervals was 95%. Next, a set for every intervention group patient was selected from the control group based on the propensity score matching obtained from the logistic regression. The model was built based on a sample of patients propensity score matched 01:01, with no replacement or repetition. Sixty-one adequately matching pairs of patients were identified, which was enough to perform all statistical analyses, without compromising the power of the study. The matching process was done before the analysis of the study results. One of the patients from the CVR group was removed from the analysis due to an inconsistency in the long-term follow-up data. Differences were considered statistically significant for *P*<0.05. All analyses were performed using IBM - SPSS (version 21, IBM Corp Armonk, NY).

## RESULTS

The group of patients who underwent aortic root reconstruction with AVS was younger. Proportionally, AVS was the most performed procedure in patients with Marfan Syndrome and it was not the technical option for reoperation. After the propensity score matching, there were no differences between groups in frequency of sex, degree of aortic regurgitation, and diagnosis of the underlying disease. Most of the patients were functional class I and II, with moderate and severe aortic insufficiency respectively at 73.3% and 87.9% in the CVR group and 78.4% and 62% in the AVS group. There were no differences in distribution between groups for the remaining variables analyzed (Table 1).

### Procedure performed (Table 2)

There were significant differences in the time needed to perform both aortic root reconstruction procedures (*P*<0.001); with CPB and myocardial ischemia times in minutes for CVR and AVS groups being 132±29 and 100±22 versus 158±31 and 135±25, respectively.

There was no difference between the sites of the arterial line, whose placement was in accordance with the underlying disease.

Seventy-six associated procedures were performed (in 23.4% of the patients), with a prevalence of myocardial revascularization (12.6%) followed by mitral valve procedures (9%).

### Hospital mortality and immediate postoperative complications

In terms of incidence of postoperative complications as well as 30-day and hospital mortality, there were no significant differences, regardless of the aortic root reconstruction technique employed, as stated in Table 3.

**Table 3:** Intrahospital postoperative complications of patients from the original CVR and AVS groups and propensity score matched groups.

	Original cohort				Propensity score matched cohort		
	CVR	AVS	*P* value		CVR	AVS	*P* value
Variables	n=263	n=61			n=60	n=61	
Reoperation, n (%)							
Bleeding	19 (97.2)	4 (6.6)	0.85		1 (1.7)	4 (6.6)	0.36
Tamponade	6 (2.3)	0 (0)	0.59		1 (1.7)	0 (0)	0.49
Gauze removal	3 (1.1)	2 (3.3)	0.23		0 (0)	2 (3.3)	0.49
							
Low cardiac output, n (%)	27 (10.3)	1 (16)	0.39		3 (5)	1 (16)	0.36
Wound infection, n (%)	32 (12.2)	4 (6.6)	0.26		8 (13.3)	4 (6.6)	0.24
Mediastinitis, n (%)	2 (0.8)	0 (0)	0.99		0 (0)	0 (0)	-
Tracheobronchitis, n (%)	13 (4.9)	2 (3.3)	0.57		1 (1.7)	2 (3.3)	0.99
Pneumonia, n (%)	59 (22.4)	7 (11.5)	0.06		11 (18.3)	7 (11.5)	0.28
UTI, n (%)	9 (3.4)	1 (1.6)	0.69		2 (3.3)	1 (1.6)	0.54
Sepsis, n (%)	31 (11.8)	4 (6.6)	0.35		3 (5)	4 (6.6)	0.99
OTI > 72h, n (%)	3 (1.1)	0 (0)	0.99		0 (0)	0 (0)	-
ARF, n (%)	34 (12.9)	7 (11.5)	0.75		3 (5)	7 (11.5)	0.32
Dialytic ARF, n (%)	10 (3.8)	0 (0)	0.21		1 (1.7)	0 (0)	0.49
Psychomotor agitation, n (%)	8 (3)	1 (16)	0.99		1 (1.7)	1 (16)	0.99
Delirium, n (%)	4 (1.5)	0 (0)	0.99		0 (0)	0 (0)	-
Seizure, n (%)	5 (1.9)	0 (0)	0.58		0 (0)	0 (0)	-
CVA (deficit), n (%)	4 (1.5)	1 (16)	0.99		0 (0)	1 (16)	0.99
CVA (transiente), n (%)	2 (0.8)	0 (0)	0.99		0 (0)	0 (0)	-
Coma, n (%)	2 (0.8)	0 (0)	0.99		0 (0)	0 (0)	-
AMI, n (%)	3 (1.1)	1 (16)	0.56		0 (0)	1 (16)	0.99
Atrial arrhythmia, n (%)	54 (20.5)	9 (14.8)	0.37		14 (23.3)	9 (14.8)	0.22
Ventricular arrhythmia, n (%)	7 (2.7)	0 (0)	0.35		1 (1.7)	0 (0)	0.49
Hospital death, n (%)	29 (11)	3 (4.9)	0.23		4 (6.7)	3 (4.9)	0.71

CVR=composite graft-valve replacement; AVS=aortic valve-sparing operation; SD=standard deviation; UTI=urinary tract infection; OTI=orotracheal intubation; ARF=acute renal failure; CVA=cerebrovascular accident; AMI=acute myocardial infarction

There was 25% respiratory tract infection; 19.4% atrial arrhythmia (all reverted before hospital discharge); 15.7% postoperative renal dysfunction at some degree, of which 19.6% needed dialysis; 11.1% surgical wound infection (superficial); 10.5% reoperation resulting from bleeding; and 8.3% neurological complication of any kind.

Mortality in 30 days was 8.3% and hospital mortality was 9.9%.

### Late evaluation of aortic valve function and heart failure

The last echocardiographic study performed during late follow-up period was carried out in 247 patients (84.6% of the general sample and 88% of the AVS group) and showed similar intensity of regurgitation between the two groups when the absence of aortic insufficiency, traces, and discrete regurgitation are taken into consideration, reaching 100% and 84.6% in the CVR and AVS groups, respectively.

While in the preoperative period, patients of the CVR and AVS groups had moderate to severe aortic regurgitation at 78.4% and 62% of the sample, respectively. The last echocardiography showed 0% and 15.4% (5.7% of which was severe regurgitation), respectively.

In the last clinical evaluation, 89.4% of the CVR group patients had FC I and II heart failure against 94% of the AVS patients.

### Late mortality and complications associated with the performed operation (Table 4)

**Table 4:** Data from late postoperative period of patients from the original CVR and AVS groups and propensity score matched groups.

	Original cohort				Propensity score matched cohort		
	CVR	AVS	*P* value		CVR	AVS	*P* value
Variables	n=263	n=61			n=60	n=61	
Thromboembolic complications, n (%)	8 (3.1)	2 (3.3)	0.99		4 (6.7)	2 (3.3)	0.43
Hemorrhagic complications, n (%)			<0.001				0.006
no	216 (82.1)	61 (100)			48 (80)	61 (100)	
minor	11 (4.2)	0 (0)			2 (3.3)	0 (0)	
major	36 (13.7)	0 (0)			10 (16.7)	0 (0)	
Endocarditis, n (%)	6 (2.3)	1 (16)	0.99		1 (1.7)	1 (16)	
Late reoperation, n (%)	14 (5.3)	1 (16)	0.32		3 (5)	1 (16)	
							
Days to the last echo, median (25% - 75%)	933 (342-2049)	1545 (611-2555)	0.01		2050 (529-2841)	1545 (611-2555)	0.76
LVEF, % (mean±SD)	64±12	61±8	0.17		61±8	60±7	0.76
LVEDV, mL (mean±SD)	127±48	158±80	0.03		140±35	158±80	0.39
							
Late heart failure, n (%)	(n=186)	(n=51)	0.134		(n=47)	(n=51)	0.43
FC I	76 (40.9)	24 (47.1)			13 (27.7)	24 (47.1)	
FC II	84 (45.2)	24 (47.1)			29 (61.7)	24 (47.1)	
FC III	20 (10.8)	3 (5.9)			4 (8.4)	3 (5.9)	
FC IV	6 (3.2)	0 (0)			1 (2.1)	0 (0)	
							
Function of aortic valve, n (%)	(n=195)	(n=52)	<0.001		(n=28)	(n=52)	<0.001
Normal	152 (77.1)	12 (23.1)			22 (81.5)	12 (23.1)	
Minimal AI	19 (9.7)	5 (9.6)			3 (10.7)	5 (9.6)	
Mild AI	20 (10.3)	27 (51.9)			3 (10.7)	27 (51.9)	
Moderate AI	1 (0.5)	5 (9.6)			0 (0)	5 (9.6)	
Severe AI	3 (1.5)	3 (5.8)			0 (0)	3 (5.8)	
							
Follow-up time, median (25% - 75%)	902 (213-1757)	1492 (487-2385)	0.05		1637 (578-2617)	1492 (487-2385)	0.51
Death 30 days, n (%)	24 (9.2)	3 (4.9)	0.44		3 (5)	3 (4.9)	0.99
Death during follow-up, n (%)	60 (22.8)	5 (8.2)	0.01		20 (33.3)	5 (8.2)	0.001

CVR=composite graft-valve replacement; AVS=aortic valve-sparing operation; LVEF=left ventricle ejection fraction; LVEDV=left ventricle end diastolic volume; FC=functional class (NYHA); AI=aortic insufficiency

During the aforementioned follow-up period, a significant difference was observed in the incidence of major hemorrhagic complications (*P*=0.006) whereas no differences between groups were observed for minor hemorrhagic complications, survival free of thromboembolic events, endocarditis, reoperation, ventricular function, and left ventricular end-diastolic volume.

Reoperations in the CVR group had to be performed in 14 patients, five of which died (35.7%). There were four composite graft-valve replacements due to endocarditis (two deaths); five stent grafting of the descending aorta (one death) and one interposition of the polyester conduit (one death), all due to an aneurysm in the descending aorta; two thoracoabdominal aorta replacements (one death); two abdominal aortic corrections and one myocardial revascularization. The only reoperation in the AVS group was an aortic valve replacement due to severe regurgitation four years after the initial operation.

**Fig. 1A:**
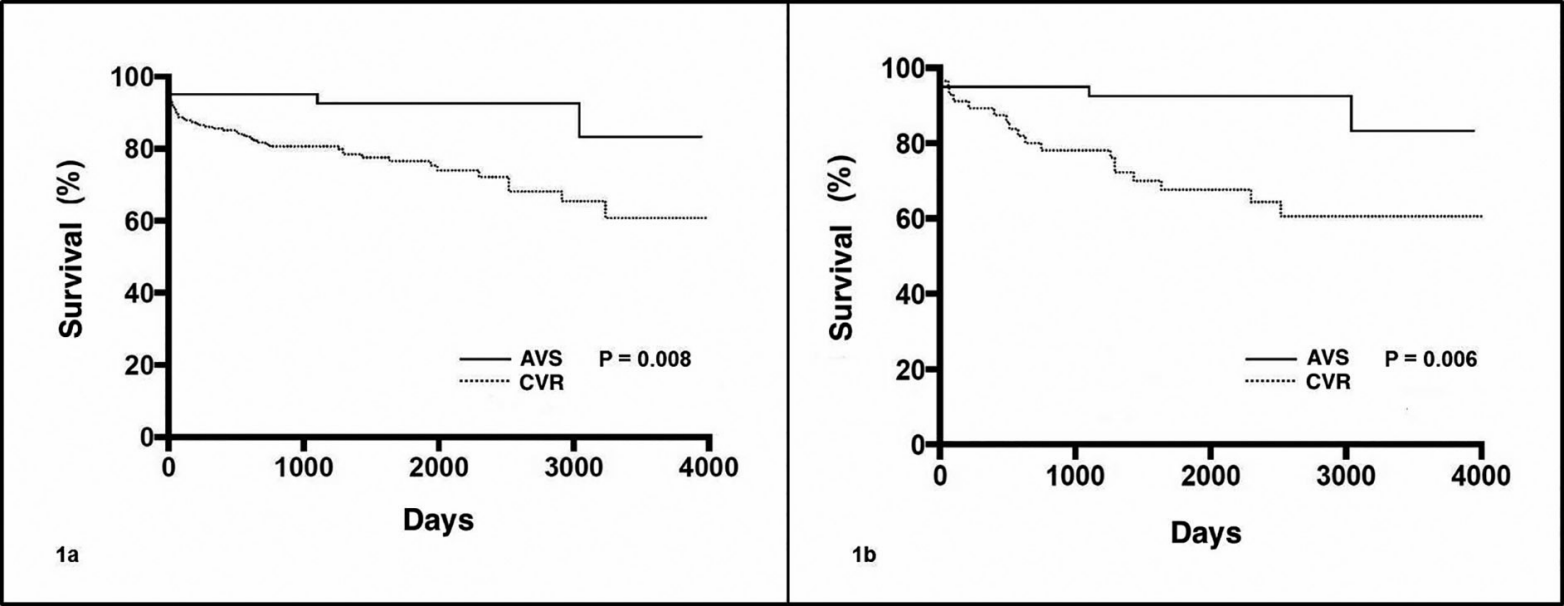
Survival curve of patients who underwent aortic root reconstruction via aortic valve-sparing operation (AVS) and composite graftvalve replacement (CVR) techniques. 1B - Survival curve of propensity score matched patients who underwent aortic root reconstruction.

Mortality during follow-up was higher in the CVR group (*P*=0.001). Looking at the survival curve, the benefit of aortic root reconstruction with AVS becomes evident (Figures 1A and 1B).

## DISCUSSION

The analysis of the results through propensity score matching allows the assessment of similar samples of patients, which would not be possible any other way since the CVR procedure is the choice of treatment for all patients and the AVS procedure is an option for selected patients, thereby making it difficult to perform a comparative analysis of both aortic root reconstruction techniques. However, there is a bias even with the use of this methodology and study groups usually are not as similar as they would be in randomized studies.

Despite good results being shown by the use of aortic valve-sparing procedures, the most appropriate procedure for aortic root reconstruction is still the composite-graft valve replacement^[9-12]^, especially because it can be performed in every single patient and it is widely applied by surgeons.

Mortality rates observed for elective patients who underwent aortic root reconstruction was 6.7% and 4.9% for CVR and AVS, respectively, which is slightly higher than the 2.9% observed in a systematic review of patients who had the aortic valve preserved and, at the same time, similar to the number of deaths among patients who underwent associated procedures (24.5%)^[9]^.

The surgical technique adopted is similar to the one used in centers where aortic valve-sparing procedures are performed. Different options adopted for the arterial line were due to the extent of the disease in the ascending aorta/aortic arch, whether the brachiocephalic trunk was compromised (in chronic dissections), and the deliberate use of the right subclavian artery for acute aortic dissections with impairment of proximal segments.

In this study, the comparative analysis showed surgical results were entirely similar between the groups, both for 30-day and overall hospital mortality. In spite of the greater complexity of the AVS procedure, clearly reflected on longer CPB and myocardial ischemia times, the immediate result of the procedure was entirely comparable. Why do it, then?

Late follow-up of these patients showed evidence of the benefits of preservation of the aortic valve with a direct impact on mortality, especially as a result of the lack of prolonged use of oral anticoagulants and the control of adequate levels of anticoagulation. Bleeding had a direct influence on the mortality of these patients (there were two cases of cardiac tamponade, three cerebrovascular accidents, one medullary vascular accident, six upper gastrointestinal bleedings, two cases of enterorrhagia, and one case of epistaxis with hemorrhagic shock). There were other bleeding events, however, without repercussions. In a systematic review of when there was preservation of the aortic valve, the bleeding observed during evolution is not cause worrying^[13]^, differently from what is observed with prolonged use of anticoagulants.

There was no significant difference in the incidence of thromboembolic and infectious complications when a valve prosthesis was used or when the native valve was spared, different from some literature citations and even from results previously observed in the present institution for patients treated at different points in time when there was a higher need for reoperation due to endocarditis of the valved conduit compared to the native valve^[4,12]^.

The need for reoperation during evolution of both groups was low. In the case of the CVR group, it was particularly as a result of the incidence of vascular disease in distal segments of those treated initially, followed by prosthetic infection. In this sample, there was no reoperation due to pseudoaneurysm. In the AVS group, average follow-up time was 1492 days and there was only one patient who needed valve replacement (1.7%); two others, despite severe regurgitation (5.1%), were asymptomatic and had neither significant dilation of the left chambers nor worsening of ventricular function and thus continued with clinical follow-up. Therefore, in aortic root reconstructions, one patient needed aortic valve replacement (1.7%) for median follow-up time and for follow-up times of 25% and 75% of the sample of 4.1 years, 1.3 years, and 6 years, respectively. Two other patients (5.1%) showed severe regurgitation in up to six years of follow-up.

Based on the information aforementioned, we suggest a reevaluation of the aortic root reconstruction via composite-graft valve replacement as the gold standard for treatment of aortic root diseases, mainly if these results remain constant in the coming years. This is in accordance with the suggestion of centers of excellence for the treatment of this subgroup of patients^[12-16]^.

### Limitations of the study

It has the limitations of being a retrospective study carried out with infrequent disorders performed in a reduced number of patients by only two surgeons and with limited follow-up time.

## CONCLUSION

Aortic root reconstruction with preservation of the aortic valve should be the procedure carried out in patients with diseases in this segment of the aorta since it has lower morbimortality and survival free of hemorrhagic events associated with prolonged anticoagulation.

**Table:** 

**Authors’ roles & responsibilities**	
FAL	Analysis and interpretation of the data
RRD	Analysis and interpretation of data; final approval of the manuscript; study design; implementation of projects and experiments; manuscript writing or critical review of its content
JAD	Analysis and interpretation of the data
LBF	Analysis andr interpretation of the data
LMSM	Statistical analysis
LFB	Manuscript writing and critical review of its content
CM	Manuscript writing and critical review of its content
FBJ	Manuscript writing and critical review of its content
